# Which are best for successful aging prediction? Bagging, boosting, or simple machine learning algorithms?

**DOI:** 10.1186/s12938-023-01140-9

**Published:** 2023-08-29

**Authors:** Razieh Mirzaeian, Raoof Nopour, Zahra Asghari Varzaneh, Mohsen Shafiee, Mostafa Shanbehzadeh, Hadi Kazemi-Arpanahi

**Affiliations:** 1 Department of Health Information Management, Shahrekord University of Medical Sciences, Shahrekord, Iran; 2 Student Research Committee, School of Health Management and Information Sciences Branch, Iran University of Medical Sciences, Tehran, Iran; 3 Department of Computer Science, Faculty of Mathematics and Computer, Shahid Bahonar University of Kerman, Kerman, Iran; 4 Department of Nursing, Abadan University of Medical Sciences, Abadan, Iran; 5 Department of Health Information Technology, School of Paramedical, Ilam University of Medical Sciences, Ilam, Iran; 6 Department of Health Information Technology, Abadan University of Medical Sciences, Abadan, Iran

**Keywords:** Machine learning, Data mining, Quality of life, Health-related quality of life, Aged, Successful aging

## Abstract

**Background:**

The worldwide society is currently facing an epidemiological shift due to the significant improvement in life expectancy and increase in the elderly population. This shift requires the public and scientific community to highlight successful aging (SA), as an indicator representing the quality of elderly people’s health. SA is a subjective, complex, and multidimensional concept; thus, its meaning or measuring is a difficult task. This study seeks to identify the most affecting factors on SA and fed them as input variables for constructing predictive models using machine learning (ML) algorithms.

**Methods:**

Data from 1465 adults aged ≥ 60 years who were referred to health centers in Abadan city (Iran) between 2021 and 2022 were collected by interview. First, binary logistic regression (BLR) was used to identify the main factors influencing SA. Second, eight ML algorithms, including adaptive boosting (AdaBoost), bootstrap aggregating (Bagging), eXtreme Gradient Boosting (XG-Boost), random forest (RF), J-48, multilayered perceptron (MLP), Naïve Bayes (NB), and support vector machine (SVM), were trained to predict SA. Finally, their performance was evaluated using metrics derived from the confusion matrix to determine the best model.

**Results:**

The experimental results showed that 44 factors had a meaningful relationship with SA as the output class. In total, the RF algorithm with sensitivity = 0.95 ± 0.01, specificity = 0.94 ± 0.01, accuracy = 0.94 ± 0.005, and *F*-score = 0.94 ± 0.003 yielded the best performance for predicting SA.

**Conclusions:**

Compared to other selected ML methods, the effectiveness of the RF as a bagging algorithm in predicting SA was significantly better. Our developed prediction models can provide, gerontologists, geriatric nursing, healthcare administrators, and policymakers with a reliable and responsive tool to improve elderly outcomes.

## Introduction

Aging is a global phenomenon that represents a significant risk factor for disability and many chronic diseases. This period of human life is a continuous but irreversible process with a steady deterioration in body structure and functions [1, 2]. Population aging will increase healthcare costs, resulting in a huge medical burden and severe financial pressure on families, which poses profound economic, political, and social outcomes for both developed and developing countries [3, 4]. The global proportion of older people aged ≥ 60 is increasing rapidly compared to other age groups [5]. Currently, it is estimated that 12.7% of the world’s population is elderly. By 2050, the elderly population is projected to make up more than 21.4% of the world’s population, and by 2100 this population will triple to reach approximately 27.7% [6]. Reports indicated that the population in Iran is in transitioning from youth to old age. About 10% of Iran's population is aged 60 years and older. According to official reports, people aged 65 and older will account for 31% of the total Iranian population by 2050, and this proportion will increase dramatically [7, 8].

In recent decades, advances inmedicine have significantly reduced global mortality rates, leading to an increase in the world’s elderly population. Aging is not a disease, but neglect of people’s health monitoring has negative impacts on all countries’ healthcare, the economy, education, employment, social, and political sectors. The negative effects of the increasing aging population include decreased quality of life (QoL), increased dependence on others for doing daily activities and mental health problems, existing problems such asloss of job, loss of spouse and friends, loss of  children, poverty, and physical problems [9, 10]. On the other hand, improving life expectancy leads to an increase in the elderly population along with the amount of time spent as an older adult. In this situation, the epidemiology of diseases among the elderly also changes to chronic non-communicable diseases such as cardiovascular diseases (CVD), hypertension, diabetes, neoplasm, and dementia. As a result, it causes social and economic problems for the elderly, so the elderly population requires more health services than other age groups [11].

The concept of successful aging (SA) emerged in the gerontological literature to overcome the challenges and problems of population aging. SA as a preferred term overlaps with various terms such as positive aging, aging well, productive aging, and healthy aging [12, 13]. The SA stressed the quality of the aging life. This paradigm shifts the focus on aging from normal aging with four Ds (disease, disability, death, and dementia) to SA assesses how people can age well, and identifies the involved processes and components with criteria to “how long,” “how well,” or “how healthy” live [14–16]. This concept has long intrigued academics and researchers. Robert Havighurs first defined SA in 1961 as feeling life satisfaction and happiness during the latter stages of an individual’s lifespan [17]. Rowe and Kahn [18] state that SA is not suffering from chronic diseases, but consists of a combination of three components, which are the low probability of disease and disease-related disability, high cognitive and physical functioning, and active engagement with life [18–20]. However, Rowe and Kahn’s theory ignored the dimension of mental health. In recent years, an increasing number of researchers have improved on the Rowe and Kahn model. For example, Crowther added “positive spirit” as a fourth dimension, and Bowling added “subjective well-being” [21].

Previous studies have mostly described factors influencing SA. However, due to the subjective, interdisciplinary, and multidimensional nature of SA, measuring or predicting is a difficult task. A fundamental emphasis of studies is on better understanding and defining SA and recognizing its determinants so that clinical care and protective interventions can be more meaningfully informed [22]. The influencing factors of SA are interdependent and complex, and the traditional model does not apply to SA [11, 23, 24]. Rapid technological and digital advancement, such as artificial intelligence (AI), provides new ways to create novel smart services or renew health pathways by lean operations [11, 25, 26]. As a subcategory of AI, machine learning (ML) is an extensive discipline based on statistics or computational science that provides automated learning techniques to extract hidden patterns from empirical data and then make complex decisions based on learned behaviors [11, 21, 27]. The present study aimed to develop several ML predictive models for predicting SA using important features that influence SA. Finally, the performance of the ML models was compared to select the best one.

## Results

### Features extraction

After the literature review, an electronic checklist was prepared based on the 102 items extracted from the literature search. In the first phase of Delphi, 55 items were rejected and 15 items were qualified for the second Delphi phase. In the second phase, 13 items were accepted and 2 items were rejected by the experts’ panel. At the end of the Delphi phase, 44 eligibility features have entered the final checklist to predict SA.

### Sample characteristics

Finally, 1465 cases participated in this study for data analysis including the 746 and 719 associated with non-SA and SA classes, respectively. The 566 and 899 cases pertained to men and women, respectively, with an average age of 68.3 ± 3.325 years.

### Multi-variable statistical analysis

The results of data analysis pertained to the SA and non-SA elderly cases using the BLR as multi-variables statistical analysis are presented in Table 1.

**Table 1:** Results of correlation of factors affecting SA

Variable name	Correlation (*β*)	Odd ratio	95% confidence interval (CI)	*P*-value
Age (years)	0.12	1.77	[1.52–1.94]	0.016*
Sex	0.18	2.45	[2.1–2.86]	0.3
Educational level	0.25	1.97	[1.68–2.2]	0.6
Marital status	0.16	0.55	[0.38–0.75]	0.21
Occupation	0.21	1.12	[0.98–1.35]	0.17
Income level	0.44	2.34	[2.12–2.76]	0.001*
Insurance status	0.32	1.69	[1.42–1.96]	0.3
Hypertension	0.37	1.71	[1.25–2.08]	0.005*
CVA	0.20	1.13	[0.98–1.32]	0.028*
Bone disease	0.1	1.05	[0.85–1.2]	0.001*
Renal disease	0.16	0.65	[0.42–0.83]	0.2
Liver disease	0.12	0.78	[0.52–0.96]	0.011*
Muscle disease	0.19	1.66	[1.45–1.9]	0.001*
Depression	0.27	1.71	[1.52–1.86]	0.013*
Convalescences	0.26	1.12	[0.89–1.36]	0.024*
Eye diseases	0.29	0.85	[0.63–1.02]	0.005*
Diabetes	0.27	1.48	[1.35–1.52]	0.001*
Cancer	0.28	1.77	[1.45–1.82]	0.001*
Other diseases	0.17	0.56	[0.32–0.78]	0.2
Sport activities	0.4	1.97	[1.75–2.23]	0.001*
Exercise time	0.38	2.39	[2.14–2.56]	0.001*
Type of exercise	0.48	2.47	[2.25–2.7]	0.001*
Sexual health	0.13	0.56	[0.35–0.89]	0.001*
Perform disease prevention activities	0.11	2.45	[2.13–2.68]	0.001*
Nutritional status	0.33	0.84	[0.55–1.23]	0.001*
Mal-nutritional status	0.20	1.24	[1.05–1.42]	0.001*
Physical activity and exercise	0.37	2.39	[2.11–2.63]	0.001*
General health	0.42	2.22	[1.98–2.43]	0.013
Pain assessment	0.07	0.62	[0.51–0.83]	0.16
Fatigue	0.21	2.27	[2.15–2.41]	0.001*
Physical dysfunction	0.44	0.75	[0.6–0.93]	0.001*
Physical function	0.47	0.69	[0.45–1.1]	0.001*
Mental disorder	0.09	1.23	[1.12–1.46]	0.001*
Physiological disorder	0.15	1.77	[1.6–1.94]	0.001*
Life satisfaction	0.11	1.7	[1.57–1.89]	0.001*
Tension management	0.44	1.93	[1.78–2.13]	0.001*
Self-efficacy	0.15	2.02	[1.97–2.16]	0.001*
Self-esteem	0.41	1.24	[1.12–1.41]	0.01*
Hope	0.43	0.74	[0.49–1.02]	0.015*
Futurity	0.38	0.63	[0.51–0.87]	0.001*
Satisfaction with social support	0.27	1.78	[1.55–2.01]	0.01*
Social functional	0.17	1.91	[1.74–2.03]	0.01*
Social and interpersonal relationships	0.36	1.77	[1.63–1.86]	0.01*
Family support	0.15	1.45	[1.27–1.6]	0.01*

In this table, the odd ratio shows the probability of occurrence of each state of variables, the CI is 95% of the occurrence of the odd ratio, and the correlation is defined as the correlation of each variable with the output class. To obtain the best influencing factors for the SA, we considered the *P* < 0.05 for these variables. In contrast, the variables with *P* > 0.05 were excluded from this study. Based on the information given in Table 1, the determinant factors of age [CI = 1.52–1.94] (*β* = 0.12), income level [CI = 2.12–2.76] (*β* = 0.44), hypertension [CI = 1.25–2.08] (*β* = 0.35), CVA [CI = 0.98–1.32 (*β* = 0.2), bone disease [CI = 0.85–1.2] (*β* = 0.1), liver disease [CI = 0.52–0.96] (*β* = 0.12), muscle disease [CI = 1.45–1.9] (*β* = 0.19), depression [CI = 1.52–1.86] (*β* = 0.25), convalescences [CI = 0.89–1.36] (*β* = 0.26), eye disease [CI = 0.63–1.02] (*β* = 0.29), diabetes [CI = 1.35–1.52] (*β* = 0.27), cancer [CI = 1.45–1.82] (*β* = 0.25), sports activities [CI = 1.75–2.23] (*β* = 0.4), exercise time [CI = 2.14–2.56] (*β* = 0.38), type of exercise [CI = 2.25–2.7] (*β* = 0.48), sexual health [CI = 0.35–0.85] (*β* = 0.13), perform disease prevention activities [CI = 2.13–2.68] (*β* = 0.11), nutritional status [CI = 0.55–1.33] (*β* = 0.33), mal-nutritional status [CI = 1.05–1.42] (*β* = 0.2), physical activity and exercise [CI = 2.11–2.63] (*β* = 0.37), general health [CI = 1.98–2.43] (*β* = 0.42), fatigue [CI = 2.15–2.41] (*β* = 0.21), physical dysfunction [CI = 0.6–0.93] (*β* = 0.44), physical function [CI = 0.45–1.1] (*β* = 0.47), mental disorder [CI = 1.12–1.46] (*β* = 0.09), physiological disorder [CI = 1.6–1.94] (*β* = 0.15), life satisfaction [CI = 1.57–1.89] (*β* = 0.11), tension management [CI = 1.78–2.13] (*β* = 0.44), self-efficacy [CI = 1.97–2.16] (*β* = 0.15), self-esteem [CI = 1.12–1.41] (*β* = 0.41), hope [CI = 0.49–1.2] (*β* = 0.43), futurity [CI = 0.51–0.87] (*β* = 0.38), satisfaction with social support [CI = 1.55–2.01] (*β* = 0.27), social functions [CI = 1.74–2.03] (*β* = 0.17), social and interpersonal relationships [CI = 1.63–1.86] (*β* = 0.36), and family support [CI = 1.27–1.6] (*β* = 0.15) obtained the correlations with the output class at *P* < 0.05. The variables including sex (*P* = 0.3), marital status (*P* = 0.21), educational level (*P* = 0.6), occupation (*P* = 0.17), insurance status (*P* = 0.3), renal disease (*P* = 0.2), other diseases (*P* = 0.2), and pain assessment (*P* = 0.16) were excluded from this study.

### Appraising the ML algorithms’ performance

The results of the evaluation metrics of ML algorithms including bagging, boosting, and simple algorithms with fivefold cross-validation are shown in Table 2.

**Table 2:** Performance evaluation of selected algorithms

Algorithm	Hyper-parameters	Sensitivity	Specificity	Accuracy	*F*-score
RF	Maximum of depth = “6” Maximum number of iterations = “50” Number of execution slots = “1” Bag size percent = “100” Break Tie randomly = “True”	0.95 ± 0.01	0.94 ± 0.01	0.94 ± 0.005	0.94 ± 0.003
Bagging	Bag size percent = “100” Classifier = “REP-Tree” Maximum number of iterations = “15” Number of decimal places = “2” Number of execution slots = “1”	0.84 ± 0.02	0.84 ± 0.01	0.84 ± 0.01	0.84 ± 0.02
AdaBoost	Batch size = “100” Classifier = “Decision Stump” Maximum number of iterations = “20” Weight threshold = “50” Minimum number of instances per leafs = “1”	0.88 ± 0.01	0.86 ± 0.02	0.88 ± 0.01	0.86 ± 0.01
XG-Boost	Maximum depth = “8” Classifier = “Decision Stump” Base score = “4” Min child weight = “1” Booster = “gb-tree”	0.90 ± 0.01	0.88 ± 0.02	0.89 ± 0.015	0.88 ± 0.01
MLP	Number of hidden layers “10” Learning rate = “0.25” Momentum = “0.2” Validation threshold = “20” Maximum number of iterations = “20” Normalize numeric values and attributes = “True”	0.77 ± 0.03	0.75 ± 0.04	0.76 ± 0.035	0.76 ± 0.035
SVM	Kernel type = “RBF” Regularization parameters (*C*) = “10” Gamma = “10” RBF gamma = “0.1” Degree for increasing dimensions = “3”	0.80 ± 0.03	0.79 ± 0.03	0.79 ± 0.03	0.79 ± 0.03
J-48	Confidence factor = “0.2” Minimum number of objects = “2” Number of folds = “3” Binary split = “false” Reduced error pruning = ” True”	0.72 ± 0.04	0.72 ± 0.04	0.70 ± 0.04	0.72 ± 0.04
NB	Use Kernel Classifier = “true” Use Supervise discretization = “true” Batch size = “100” Number of decimal places = “100”	0.68 ± 0.04	0.65 ± 0.05	0.69 ± 0.045	0.66 ± 0.04

**Fig. 1:**
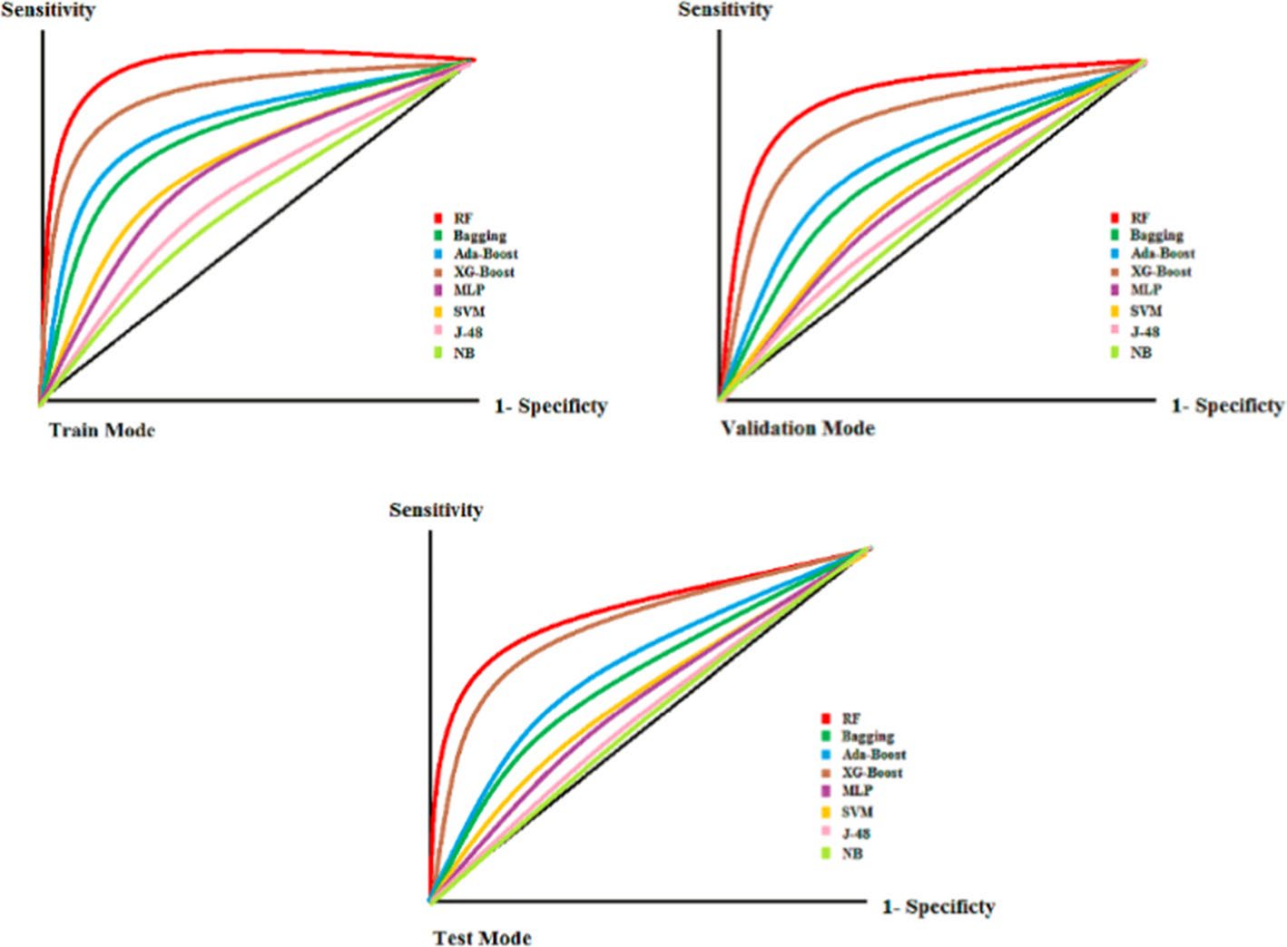
The ROC of all statutes of ML algorithms

Based on the evaluation metrics presented in Table 2, the RF model by the maximum tree depth of 6 and 50 of algorithm’s iteration with sensitivity = 0.95 ± 0.01, specificity = 0.94 ± 0.01, accuracy = 0.94 ± 0.005, and *F*-score = 0.94 ± 0.003 gained the best predictive strength in classifying the SA and non-SA cases among older adults. The XG-Boost-trained algorithm with decision stump as a base classifier and gb-tree as an objective function with sensitivity = 0.88 ± 0.01, specificity = 0.86 ± 0.02, accuracy = 0.88 ± 0.01, and *F*-score = 0.88 ± 0.01 was ranked as a second predictive performer in terms of SA compared to other ML-trained algorithms. Also the AdaBoost with the decision stump as a base classifier and maximum iteration equaled to 20 with sensitivity = 0.88 ± 0.01, specificity = 0.86 ± 0.02, accuracy = 0.88 ± 0.01, and *F*-score = 0.86 ± 0.01 and bagging-trained algorithm with sensitivity = 0.84 ± 0.02, specificity = 0.84 ± 0.01, accuracy = 0.84 ± 0.01, and *F*-score = 0.84 ± 0.02 got the third and fourth predictive strength ranks among other ML-trained algorithms. Investigating the predictive strength of these four ensemble ML-trained algorithms using the mentioned performance indicators in this study showed that all of them obtained the pleasant capability in categorizing the SA and non-SA cases among the elderly with all performance criteria obtained more than 80%. The NB algorithm with sensitivity = 0.68 ± 0.04, specificity = 0.65 ± 0.05, accuracy = 0.69 ± 0.045, and *F*-score = 0.66 ± 0.04 obtained the lowest performance in this respect. With the exception of the NB algorithm, all other ML algorithms gained a performance of more than 0.7. However, the bagging and boosting algorithms gained more predictive strength in SA than other simple ML-trained algorithms. The results of comparing the algorithms based on the AUC curve in train, test and validation modes are shown in Fig 1.

By assessing and comparing the performance of all bagging, boosting, and base algorithms in all train, validation, and test situation, we resulted that the RF model as a bagging algorithm with AUC-train = 0.918, AUC-validation = 0.886, AUC-test = 0.845 gained the best predictive strength to classify the SA and non-SA cases among the older adults. The XG-Boost prediction model with AUC-train = 0.893, AUC-validation = 0.865, and AUC-test = 0.832 obtained the second predictive capability in classifying these cases as a boosting method. Also, the test results obtained by this algorithm showed the pleasant generalizability capability in classifying the SA and non-SA cases than RF model (we saw the less reduction of predictive power in test state result than the RF-trained ML by analyzing the ROC). Also, the AdaBoost and bagging algorithms with AUC-train = 0.836, AUC-validation = 0.765, AUC-test = 0.715 and AUC-train = 0.819, AUC-validation = 0.743, AUC-test = 0.703 gained the relative pleasant performance by AUC > 0.7 in all training, testing, and validation states. On the contrary, The J-48 and NB algorithms as the base algorithms with AUC-train = 0.623, AUC-validation = 0.558, and AUC-test = 0.531 and AUC-train = 0.569, AUC-validation = 0.526, and AUC-test = 0.512, respectively, gained the worst performance strength in this regard. In general, the evaluation of the functionality of all three types of bagging, boosting, and simple algorithms showed that the ROC values of the bagging and boosting were closer to the sensitivity vertices and so had the more favorable prediction strength for predicting the SA and non-SA cases among the elderly than the simple ML algorithms.

### Overall schema indicating the performance and external testing prediction models

**Fig. 2:**
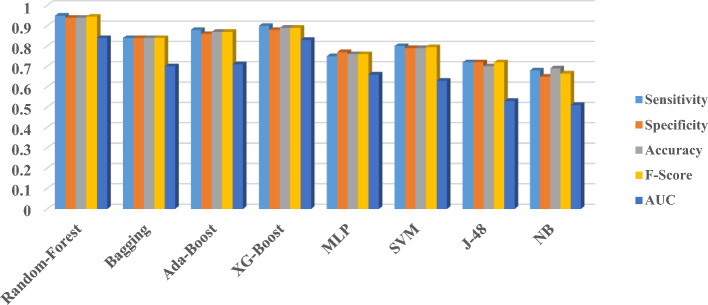
The performance criteria of bagging, boosting, and simple algorithms

An overview of all data mining algorithms’ performance results including bagging, boosting, and simple algorithms based on sensitivity, specificity, accuracy, *F*-score, and AUC-test is shown in Fig. 2.

Figure 2 shows that the RF, bagging, AdaBoost, and XG-Boost as the ensemble algorithms obtained better performance than the SVM, MLP, J-48, and NB as the simple algorithms to classify the SA and non-SA cases. The RF and XG-Boost obtained pleasant performance for classifying the SA and non-SA cases, but the RF as a bagging technique algorithm gained better performance than the two other boosting algorithms. In contrast, the NB-trained algorithm gained the worst performance in this respect. Evaluating the performance criteria considering the test state showed that RF and XG-Boost-trained algorithms with AUC-test gained the best generalizability capability than other ML-trained algorithms. Thus, these two ML-trained algorithms are more exposure to leveraging in external settings than others by pleasant performance demonstrated in test data. To evaluate the external validity of our best-trained ML algorithms we used these two models to test the predictive capability of them in predicting the external samples of SA and non-SA. We used the cases pertained to SA and non-SA cases belonged to one elderly center of the Abadan city. Also, the 45 and 70 cases associated with the SA and non-SA cases pertained to all older adults interviewed in this center were used for external evaluation. In this respect, we reported the external validity results using the confusion matrix and the ROC obtained by test data. The results of the classification of these external test cases using the confusion matrix are shown in Table 3.

**Table 3:** External test classification by models

	Forecasted as SA	Forecasted as non-SA
SA cases	38 (RF) 37(XG-Boost)	7 (RF) 8 (XG-Boost)
non-SA cases	8 (RF) 11 (XG-Boost)	62 (RF) 59 (XG-Boost)

**Fig. 3:**
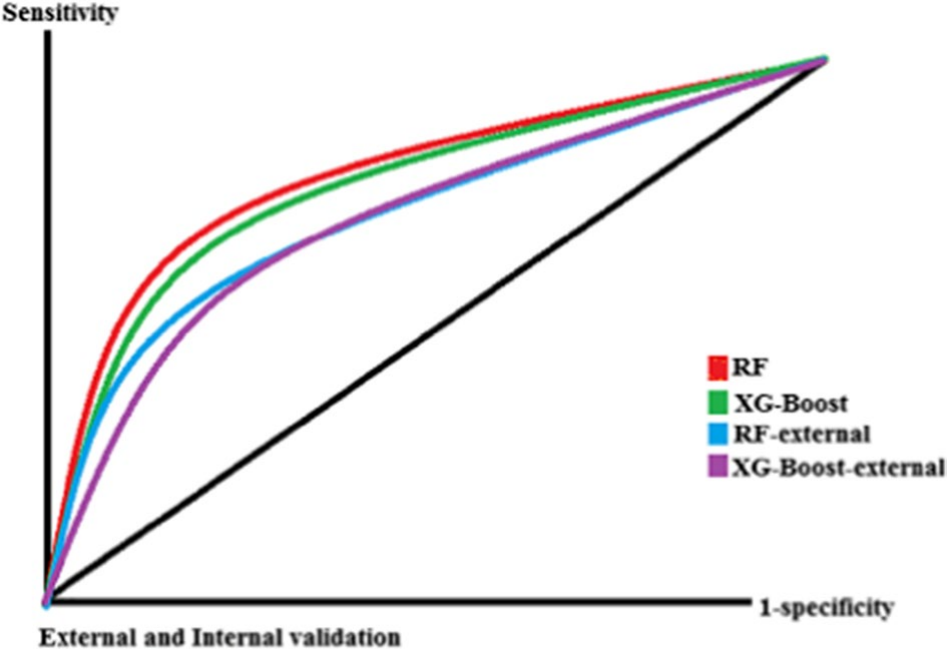
The ROC of internal and external validation

Based on the information given in Table 3, the RF and XG-Boost models gained sensitivity = 0.84, specificity = 0.88, and accuracy = 0.86, and sensitivity = 0.82, specificity = 0.84, and accuracy = 0.83, respectively. In external state comparing to these performance criteria in internal validation, we did not obtain high reduction performance capability (average reduction < 10%) by these two algorithms. Also comparing the classification capability in test and train states confirms this subject (all ROC values pertained to RF, XG-Boost, and external test modes are close to each other) (Fig. 3).

### Feature importance based on RF

**Fig. 4:**
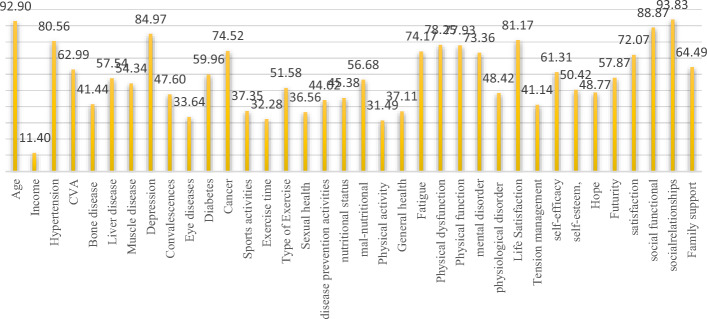
The NI of all selected variables affecting the SA

Based on the RF algorithm, the features influencing the SA are described as their importance for prediction based on the Net Importance per percent (NI%) obtained with this algorithm. This result is shown in Fig. 4.

Based on Fig. 4, the variables of age with NI = 92.9%, social functional with NI = 88.87%, social interpersonal relationship with NI = 93.83%, depression with NI = 84.97%, and hypertension with NI = 80.56% gained NI > 0.8 and were considered as the nest factors influencing the SA by the RF as the ensemble algorithm. The variable of income with the NI = 11.4% obtained the least amount in this regard. Based on the results, it is concluded that the social factors with a higher NI than other physical, demographic, and mental variables can be considered as important factors influencing SA. In other words, improving the modifiers of social factors has a potential role in increasing the SA in the elderly.

## Discussion

The aim of this study was to predict SA using ML methods. For this purpose, data of persons aged 60 years and older were analyzed. For doing this, at first, the most relevant predictors related to SA were selected by using the BLR at *P* < 0.05. Then, eight well-known and commonly used algorithms such as AdaBoost, XG-Boost, Bagging, RF, J-48, MLP, SVM, and NB were trained. Finally, several evaluation metrics derived from the confusion matrix were calculated to validate the models. Our study applied some individual implementation, bagging, and boosting ML techniques to predict SA. In our study, the RF achieved the best performance as an ensemble and bagging algorithm. This algorithm can prove the strong performance of DTs in predicting SA.

To date, little research has been performed to classify SA using ML models. Kaur et al. assessed the performance of six ML algorithms to predict the national QoL and life satisfaction. In their study, the DT model showed the best performance with a root mean square error (RMSE) of 0.3. In addition, it is recognized that various factors such as income level, underlying condition, social support and engagement, housing condition, and access to services contribute highly to the prediction of SA [28]. Lee et al. compared the performance of three common supervised ML algorithms for elderly health-related quality of life (HRQoL) with chronic diseases. Five factors with statistical significance were identified for HRQoL: monthly income, chronic disease diagnosis, depression, discomfort, and perceived health status. Finally, the DT algorithm yielded the best performance with an accuracy of 0.93 and an *F*-score of 0.49 [29]. Another study by Abdullah et al., presented a model for identifying QoL predictors based on the RF model. In this study, some variables such as lifestyle, exercise, social interaction, healthcare accessibility, chronic morbidity, and income wereproposed as the most effective predictors of QoL [21]. Sim et al. designed an intelligent clinical decision support system (CDSS) based on ML algorithms to predict HRQoL. Finally, the RF algorithm yielded the best performance with an AUC-ROC of 0.898 [30]. Cai et al. evaluated the performance of selected ML algorithms using a dataset including 3657 community-dwelling adults aged ≥ 60 years to predict SA. Finally, the DT model with an AUC of 0.90% was introduced as the most appropriate algorithm, and age, arm curl, 30-s sit-to-stand, and reaction time were introduced as important predictors in all models [11]. Paul et al. trained ensemble ML techniques to recognize ADLs in elderly people with HIV. After execution, the XG-Boost method obtained an average AUC of 83% [31]. Zhou et al. trained some ML techniques such as DT, XG-Boost, Ada boosting, bagging, and RF to classify the healthy behaviors of the elderly. Their findings showed that ensemble techniques can improve the performance of models [32]. Lee et al. compared the performance of single and ensemble ML models to predict depression in elderly people. The results showed that ensemble models increased modeling performance [33]. Lin et al. also evaluated the prediction performance of the bagging ensemble ML method with other basic ML methods such as linear regression, SVM, multilayer feedforward neural networks, and RF  to predict the functional outcomes of schizophrenia. Finally, the bagging ensemble algorithm outperformed the other techniques [34]. Ahmadi and Asghari Varzaneh in separate studies [35, 36] developed ML models for the prediction of SA. The comparison results of the experiments conducted in their studies show that the present study has evaluated a larger number of ML algorithms for predicting SA in older adults. The results of the current study showed that the use of a larger number of algorithms can lead to higher accuracy and better predictive power. However, it is important to note that the study populations, features, and predictors used in the three studies were different, which may have influenced the results. Nonetheless, our results suggest that a more comprehensive approach to SA prediction can provide valuable insights into the factors contributing to SA and improve outcomes for older adults.

Although the current study presented an optimum performance in predicting SA in older adults, it had several potential limitations and challenges. We only applied eight ML techniques on a small dataset of elderly individuals and did not use complex deep learning (DL) models due to their high data requirements. DL methods can learn complex representations of data but may overfit with small datasets due to a large number of parameters and sensitivity of optimization algorithms to available data. ML methods may be more suitable for small datasets. Although DL methods can achieve high performance, they may not be appropriate for small dataset classification. However, the accuracy and generalizability of our models will be enhanced if we test other ML techniques, as well as DL models at the larger, multicenter, and prospective dataset containing time-varying covariates to identify a more insightful set of longitudinal factors related to SA. In addition, the external validation method should be used to prove the results of the present study. Another posible limitation of this research is that it does not explain how the predictor and outcome variables are related causally. This causal relationship is not the main purpose of this research, but it is certainly suggested in future research to determine a set of longitudinal features related to SA.

In this study, ML models were developed and evaluated for predicting SA in older adults. These models have the potential to provide valuable tools for improving elderly outcomes and increasing the probability of SA. However, their practical implementation must be carefully considered, and further research is needed to validate and refine the models in different populations and settings. The potential benefits of using these models in clinical practice and policymaking are significant. They can assist geriatricians, senior nurses, healthcare administrators, and policymakers in providing optimal supportive services and customized therapeutic care for elderly persons. Additionally, the models can be used in combination with other tools and interventions to improve outcomes for older adults. However, the limitations of the models must also be acknowledged, and ethical and privacy concerns related to their use must be addressed. In future research, the models developed in our study could be applied and customized to other social problems. This could lead to a better understanding of the factors contributing to SA and help improve health outcomes and QoL for older adults. Overall, our study provides a valuable contribution to the field of SA prediction using ML, and we hope that these models will be used to benefit older adults in the real-world.

## Conclusions

The main idea of this study is to evaluate several ML models to predict SA. This study can assist geriatricians and senior nurses in providing optimal supportive services and customized therapeutic care for elderly persons by analyzing their physical, psychological, and particularly social features and extracting the best evidence from the data. Our models also have the potential to provide healthcare administrators and policymakers with a reliable and responsive tool to improve elderly outcomes. These predictive models may also provide an advantage in increasing the probability of SA. In future research, our models are expected to be applied and customized to other social problems.

## Methods

### Study design and setting

This research is a cross-sectional study that was performed in 2022. We included the data of 1465 elderly people who referred to healthy settings in Abadan City Iran. In our study, aged 60 years and older are considered the elderly. Developed countries consider the age of 65 as the onset of old age. But the United Nations and the World Health Organization (WHO) recognize 60 years and older as elderly [37, 38].

### Study roadmap

**Fig. 5:**
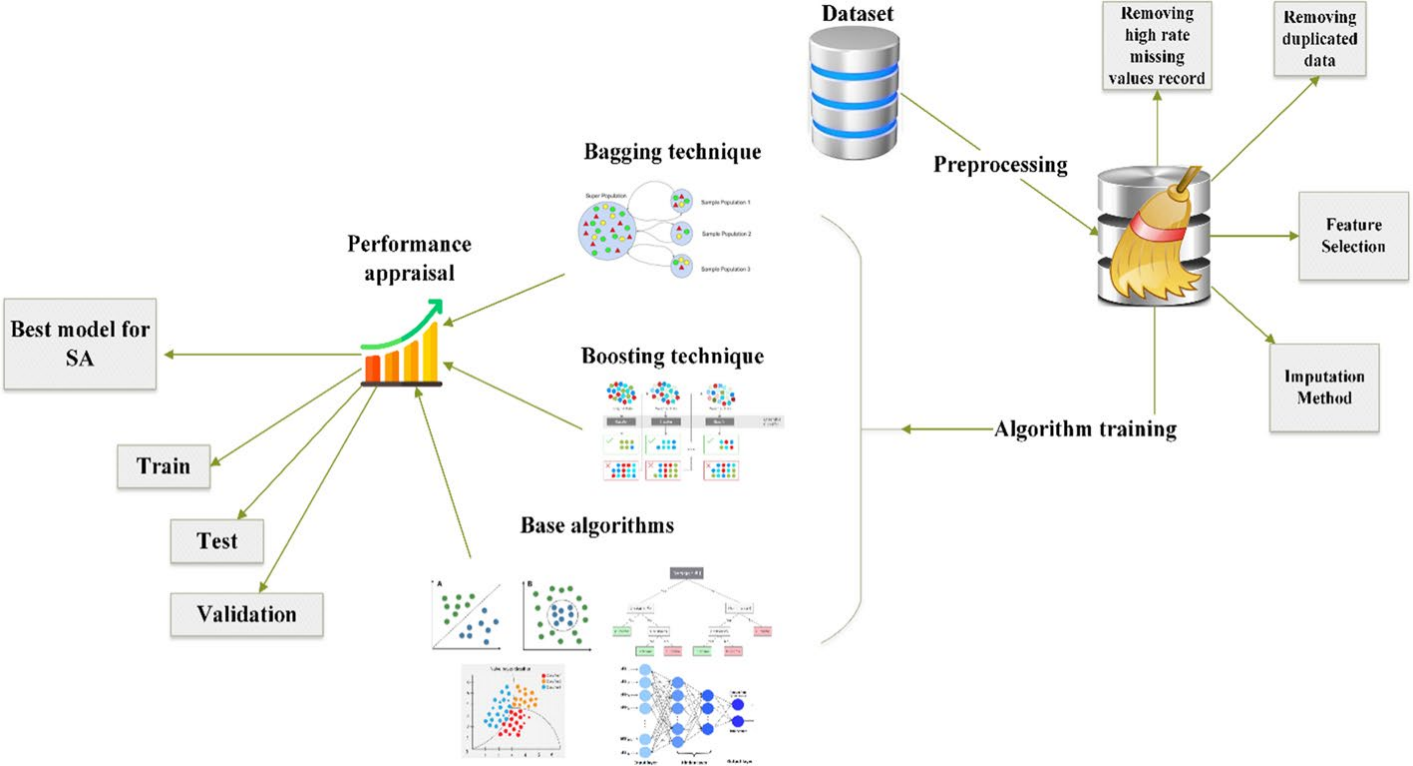
The study roadmap describing the study

This study included three phases: 1—dataset preprocessing, 2—model development, and 3—evaluating the algorithms’ performance. The roadmap of this study is depicted in Fig. 5.

### Data preparation

The SA variables are classified into socio-demographic, biomedical, and psychosocial classes. Data preparation is performed as follows:

### Primary features selection

SA is a multidimensional concept, so finding predictive factors of SA is difficult. Therefore, a comprehensive literature review was performed to extract the potential features related to SA. The primary feature set prepared in the form of a checklist and then the most important features were selected by the Delphi study.

### The panel of experts in the Delphi phase

A panel of experts, including 20 people, was contracted according to the following criteria: (1) should have knowledge related to older adults’ health; (2) have more than 5 years of experience and/or scientific publications; (3) participants must consent to participate in this study and return the checklist. First, the purpose of this study was sent to the experts through emails, and informed consent for participation was received from them. Then, the electronic checklist was emailed to them. The experts’ panel included 13 gerontologists, two geriatrics nursing, two health information management specialists, and three epidemiologists. About 52% of the participants of the Delphi stage were females, the mean of their work experience and the mean of their age were 18 ± 3.2 SD and 45.6 ± 6.4 SD, respectively.

### Predictor and outcome variables

**Socio-demographic variables:** This class includes variables such as age, gender, educational level, marital status, occupation, income level, and insurance status.

**Biomedical variables:** This class was about physiological function, cognitive function, health, and the ability to do activities of daily living (ADLs). These variables are comorbidity diseases (hypertension, cardiovascular accidents (CVA), osteopathic, eye disease, renal disease, liver disease, muscle disease, diabetes, cancer, convalescences, and other diseases), physical activity (sports activities, exercise time, type of exercise), sexual health (sexual health assessment), general health, pain assessment, fatigue, physical dysfunction, physical function, physical activity and exercise, assessment nutritional status, assessment mal-nutritional status, perform disease prevention activities, mental disorder, and physiological disorder.

**Psychosocial variables:** This class was actively engaged in life and well adapted to life including life satisfaction, tension management, self-efficacy, self-esteem, hope, futurity, social and interpersonal relationships, satisfaction with social support, and social functions.

### Definition of variables

Some variables were defined as follows:

***Ability to perform activities of daily living (ADLs):*** This variable is measured by the Barthel Index, which has 10 questions to measure physical functioning. Barthel Index determines one’s ability to perform basic ADLs, e.g., dressing, on a scale ranging from 0 to 100. Scores of 0–20 indicate severe dependence, 20–60 complete dependence, 61–90 moderate dependence, 91–99 partial dependence, and 100 indicate complete independence [33]. In this study, an independent person is someone who has a score of 100 based on the Barthel index.

***Life satisfaction:*** This variable was measured by the life satisfaction scale developed by Diener et al. [39]. This scale consisted of 5 items measuring the cognitive component of well-being. Each statement has seven options and is scored from 1 to 7 (strongly disagree to agree strongly). The validity of this instrument was confirmed by Bayani et al. [34]. In this study, a person who is satisfied with life receives a score of > 20 on this scale.

***QoL:*** The 36-Item short-form survey (SF-36) was administered to measure this variable. This self-report questionnaire consists of 36 items and eight domains: physical function, social function, physical role-playing, emotional role-playing, mental health, evaluations of vitality, physical pain, and general health. In addition to these sections, SF36 also provides two general measures of physical health [total physical component score (PCS)] and mental and social health [total mental component score (MCS)]. The respondents’ scores in each domain vary from 0 to 100, and a higher score means a better QoL. The validity and reliability of this questionnaire have been confirmed in the Iranian population [35–37].

***Physical activity, social, and interpersonal relationships:*** These factors are the SF-36 sub-categories evaluated in the elderly. In addition, the overall score was calculated to measure the QoL of the elderly. In this study, a score of 70 was considered the cut-off point for this variable.

***Healthy lifestyle:*** Lifestyle determination generally depends on the total score obtained and is calculated by getting a score of 42–98 indicating an unfavorable, 99–155 showing a medium, and 156–216 denoting a desirable lifestyle. It measures physical activity, exercise, recreation, healthy eating, stress management, and social and interpersonal relationships [38].

***Nutrition status:*** The Mini Nutritional Assessment questionnaire was administered to measure the healthy nutritional status of the elderly. In this questionnaire, a score of 12 or greater indicates that the person is well nourished and needs no further intervention. A score of 8–11 shows that the person is at risk of malnutrition. A score of 7 or less demonstrates that the person is malnourished [40]. The cut-off point of this variable in our study is 12.

***Stress management:*** The Stress Management Questionnaire was used to describe the participant’s ability to cope with difficult and stressful situations. The total scores were divided into three levels low (0–30), moderate (31–39), and high (40–50) [41]. The cut-off point of this variable in our study is 31.

***Hope:*** This factor was measured with the Hearth Hope Index tool. This tool has three characteristics of hope, including temporality and future, positive readiness and expectancy, and interconnectedness. This tool has 30 items and each item is scored between 0 and 3. A score of 3 indicates that the item applies a score of 0 indicates that the statement never applies to the respondent. Total scores can range from 0 to 90; higher scores indicate greater hope [42].

***Self-efficacy:*** Self-efficacy means the effectiveness and ability of a specific performance. The general self-efficacy (GSE) scale measured this factor. This tool has 10 items. For the GSE, the total score ranges between 10 and 40, with a higher score indicating more self-efficacy [43].

***Self-esteem:*** This factor was measured with the Rosenberg Self-Esteem Scale. This tool has 10 items and each item is scored from 1 to 4. A score of 1 indicates Strongly Disagree and a score of 4 means Strongly Agree [44, 45].

**Outcome variable (SA):** The outcome variable was categorized into SA (coded 1) or non-SA (coded 0) classes. SA can be operationally defined as the ability of individuals to maintain physical, cognitive, and social functioning as they age, while avoiding disease and disability. This can be measured using a variety of indicators, such as physical performance tests, cognitive assessments, and self-reported measures of well-being. to be considered aging successfully, individuals should score well on these indicators and demonstrate a high level of functioning across multiple domains. Importantly, SA is a multidimensional concept that encompasses physical, cognitive, and social domains and is not simply a matter of avoiding disease or disability. One common model of SA is the “three-component model” proposed by Rowe and Kahn. In our study, SA was determined based on Raw and Khan’s model which has three principal components: “absence of disease and disease-related disability,” “maintenance of high mental and physical function,” and “continued engagement with life” [40]. According to this model, in our study the following inclusion criteria of SA were used: (1) absence of disease-related disability (the criteria met in this domain are being satisfied when adults have no disability and the number of chronic diseases ≤ 2 and a score below the median on the WHODAS-II), (2) maintenance of high mental and physical function (in this domain, the participants in our study had a Mini-Mental State Examination for Dementia Screening (MMSE-DS), a score of normal and a Bartle index = 100, and no presence of depression in the previous 12 months), and (3) “continued engagement with life” (in our study, life engagement is measured using Utrecht General Engagement Scale (UGES) and participants had engaged in three or more different social or religious activities at least once a month) [24, 41–44]. All predictor variables are shown in Table 4.

**Table 4:** The elderly’s characteristics investigated in this study

Category	Variable name	Variable values	Frequency of elderly participants
Socio-demographic factors	Age (years)	60–69	813
		70–79	383
		> 80	269
	Sex	Female	922
		Male	543
	Educational level	No literacy	754
		Elementary	297
		Diploma	245
		Academic	169
	Marital status	Married	874
		Single	135
		Divorced	121
		Widowed	235
	Occupation	No job	212
		Housekeeper	624
		Retired	443
		Self-employment	186
	Income level	Under the poverty line	1121
		On the poverty line	344
	Insurance status	Yes	1114
		No	351
Biomedical factors	Hypertension	Yes	1123
		No	342
	CVA	Yes	572
		No	893
	Bone disease	Yes	894
		No	571
	Renal disease	Yes	364
		No	1101
	Liver disease	Yes	320
		No	1145
	Muscle disease	Yes	935
		No	530
	Depression	Yes	588
		No	877
	Convalescences	Yes	270
		No	1195
	Eye disease	Yes	460
		No	1005
	Diabetes	Yes	622
		No	843
	Cancer	Yes	418
		No	1047
	Other diseases	Yes	256
		No	1209
	Sports activities	Yes	971
		No	494
	Exercise time	No exercise	913
		< 30 min	383
		> 30 min	169
	Type of exercise	No exercise	912
		Aerobic	224
		Non-aerobic	143
		Both	186
	Sexual health	Unhealthy	1271
		Healthy	294
	Perform disease prevention activities	Yes	472
		No	993
	Nutritional status	Fair	951
		Good	514
	Mal-nutritional status	No	571
		Yes	894
	Physical activity and exercise	No	892
		Yes	573
	General health	Unhealthy	992
		Healthy	473
	Pain assessment	No	473
		Yes	992
	Fatigue	No	573
		Yes	892
	Physical dysfunction	No	484
		Yes	981
	Physical function	No	484
		Yes	981
	Mental disorder	No	491
		Yes	974
	Physiological disorder	No	991
		Yes	474
Psychosocial factors	Life Satisfaction	Pleasant	534
		Unpleasant	931
	Tension management	Yes	942
		No	523
	Self-efficacy	No	491
		Yes	874
	Self-esteem	No	991
		Yes	474
	Hope	No	871
		Yes	594
	Futurity	No	870
		Yes	595
	Satisfaction with social support	Pleasant	334
		Unpleasant	1131
	Social functional	No	484
		Yes	981
	Social and interpersonal relationships	Weak	399
		Strong	1066
	Family support	Yes	251
		No	1214

### Identified SA in an elderly population

Based on selected variables, a cross-sectional study was performed in this phase. People aged 60 years and older were referred to health centers in Abadan city, Iran for a check-up of their health condition. Elderly participants were selected randomly from the list of the personal health records of the health centers and clustered according to their social levels. Cluster sampling allows the researcher to identify clusters based on the different conditions of the research environment. This factor causes this study  to contain participants with different social conditions, meaning that the participants in this study were from different social levels. After determining the sample, participants were invited for an interview. The sample size in this study was 1465 people. To determine the sample size, the Cochran formula (Eq. 1) is used [46], and the sample size of the study was determined. *P* = 24% (*P* is the percentage of SA in the study of Shafiee et al. [3]) and Alpha = 1% (*α*).

1 $$n \, = \frac{{p\left( {1 - p} \right)}}{{\frac{{e^{2} }}{{z^{2} }} + \frac{{p\left( {1 - p} \right)}}{N}}} = \frac{{0.24\left( {0.76} \right)}}{{\frac{{0.05^{2} }}{{1.96^{2} }} + \frac{{0.24\left( {0.76} \right)}}{40000}}} = 279,$$

*n* = sample size; *N* = population size; *e* = acceptable sampling error; *p* = the population proportions; *z* = *z* value at reliability level or significance level; reliability level 95% or significance level 0.05; *z* = 1.96.

The objectives of this study were explained to the participants and they contributed to the study if they wished. Inclusion criteria were age ≥ 60 years, having good cognitive, and volunteering to participate in this study. Excluded criteria were as follows: participants who did not intend to cooperate, participants who had mental disorders, participants who did not have the ability to answer, people who did not have the ability to remember their past month, and people who left their interview incomplete for any reason. On the other hand, the interview was conducted by trained people that they are blinded to the purpose of this study. The researcher designed the interview process under the supervision of an epidemiologist, and questions were asked in such a way as to reduce the amount of social desirability bias. Informed consent was reviewed with each participant in a private room at the health center. Each interview took approximately 25 min, see the frequency of elderly participants in Table 4.

### Feature selection

The feature selection method was used to reduce the dataset dimension and augment the data mining performance in the third step. Feature selection in a high-dimensional dataset is one of the most important data mining steps, eliminating redundancy and irrelevant features. Feature selection is the use of statistical methods for reducing the dataset dimension. Concisely, some advantages of this process can be addressed as improving the mining performance, preventing overfitting the algorithms, increasing the computational capability, speeding up the data mining process, and increasing the understandability [47–51]. In this study, to gain the most critical factors affecting SA in the elderly, we used the binary logistics regression (BLR) as a multi-variable method to get the most important factors influencing SA. Also, the *P* < 0.05 was considered the statistically significant level in this regard.

### Model implementation

We trained eight ML algorithms using three learning methods classifications including bagging [random forest (RF) and bootstrap aggregating (Bagging)], bossing [adaptive boosting (AdaBoost) and eXtreme Gradient Boosting (XG-Boost)], and simple techniques (J-48, multilayered perceptron (MLP), Naïve Bayes (NB), and support vector machine (SVM)) in Waikato environment for knowledge analysis (WEKA) and Python programing language. In this step, the data mining process was performed using the selected algorithms because of primarily used in recent research with high-performance capability. The reason for using these algorithms is to explore the strengths and limitations of each approach and to gain insights into the factors that are most important for predicting SA. Since SA is a complex and multifaceted concept, it requires a multidimensional approach that can capture the diverse range of factors that contribute to it. By using a variety of algorithms, researchers can better understand which features and models are most effective for predicting SA and can create a more accurate and robust model. Additionally, using multiple algorithms can help reduce the risk of overfitting and increase the generalizability of the model to new data. Overall, using multiple ML algorithms can provide a more comprehensive and insightful analysis of the factors that contribute to SA. The selected algorithms in our study were described as follows:

**RF:** As an ensemble technique, RF is a bootstrap bagging technique aggregating several decision tree algorithms to enhance the algorithm’s performance. The feature with the lowest Gini Index (Eq. 2) is considered to select the best feature for data splitting:

2 $${\text{Gini}}\;{\text{Index}}\;\left( x \right) = 1 - \mathop \sum \limits_{i = 1}^{n} P\left( {x_{i} } \right)^{2} .$$

This algorithm has the strategy of voting sub-algorithms for calculating the performance. Indeed, the algorithm’s capability is the performance of most similar trees in voting in the forest. The RF algorithms are suitable for high-dimensional datasets with numerous data samples. RF is an averaging method for reducing variance using deep decision trees from different training data parts. Usually, this method slightly increases bias and a slight loss of interpretability, but it will generally significantly improve the model’s performance. In this decision tree type, the splitting process will occur using the input variables in the sub-dataset. The most prominent of this algorithm can be mentioned as a good prediction model for predicting missing data, common for working with imbalanced data for error reduction, and the importance of variables in the classification [52–54].

**Fig. 6:**
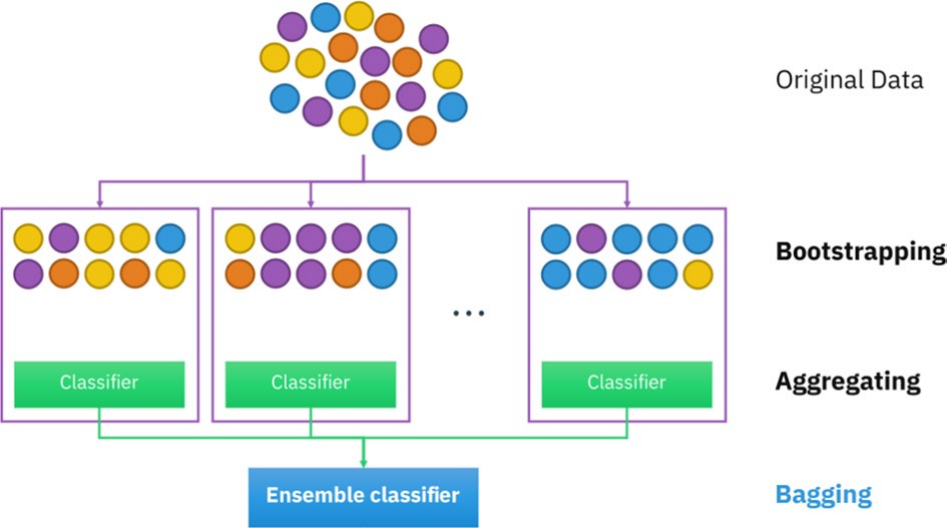
The bootstrapping method in bagging techniques

**Bagging:** Bagging is another ensemble ML algorithm using the bootstrap aggregating method during the training process. It is designed to promote the capability of the algorithms used to classify and predict cases. This algorithm uses the decision tree or other algorithms such as artificial neural networks (ANNs) or logistics regression. In the bootstrapping method (Fig. 6), the various algorithms are trained using the samples obtained by sampling with replacement. Based on the voting method, this algorithm considers the capability of classification capability pertained to most developed algorithms. One celebrated specification of this algorithm can be cited as reducing the variance and so the minimum probability of overfitting during the training process [55–57].

**AdaBoost:** the AdaBoost algorithm uses weak algorithms to predict the output class. The idea of boosting is to enhance the poorer data mining algorithms’ performance by combining them in one algorithm as a boost. This algorithm achieves the votes from the various classifier regarding the performance capability in dataset classification for better performance. So, this can provide a high computational capacity in classifying the classes. Some advantages of this algorithm can be noted as powerful acceptance in categorizing samples without any predefined knowledge existing in data, refraining from classifying the samples organized hard, and minimizing the bias and variance using the repeatable and coherent essence [58–60].

**XG-Boost:** The XG-Boost acts as a classifier and regressor in data mining. This pleasant boosting algorithm performs the prediction model using several boosted decision trees in a parallel way by gradient descent method. The enhancements of the values on the objective function (Eq. 3) are considered necessary during training the algorithm and building the boosted trees:

3 $${\text{Obj }}\left( \theta \right) = \frac{1}{n}\left( {\mathop \sum \limits_{i = 1}^{n} L\left( {y_{i} - x_{i} } \right) + \mathop \sum \limits_{j = 1}^{n} \Omega f\left( j \right)} \right) .$$

In Eq. 3, *L* is equivalent to the loss function during training the algorithm to assess the XG-Boost performance when training and ${\Omega }$ are equivalent to the regularization parameter to evaluate the algorithm’s functionality and overfitting situation. The *f*(*j*) is prediction pertained to the *j*th number of trees [61–63]. The Hessian (Eq. 4) and gradient descent functions are used to build the algorithm:

4 $$h_{{\text{m}}} \left( x \right) = \frac{{\partial^{2} L \left( {Y,g\left( x \right)} \right)}}{{\partial ,g\left( x \right)^{2} }}.$$

In Eq. 4, *g*(*x*) = *g*^m−1^(*x*), and *L* equals the loss function.

5 $${\text{Similarity}}\;{\text{Score}} = \frac{{\left( {{\text{total}}\;{\text{ residuals}}} \right)^{2} }}{{\left( {M + \lambda } \right)}}.$$

In Eq. 5, *M* and *λ* are equal to regularization parameters during the training of the algorithm.

The gain value associated with the root node is calculated as Eq. 6.

6 $${\text{Gain}} = {\text{right}}\;{\text{similarity}} + {\text{left}}\;{\text{similarity}} - {\text{root}}\;{\text{similarity}}.$$

So, the output of the algorithm is calculated as follows:

7 $${\text{target}} = \frac{{\sum {\text{Residuals}}_{i} }}{{\sum \left( {{\text{Previous}}\;{\text{possibility}}_{i} *\left( {1 - {\text{Previous}}\;{\text{possibility}}_{i} } \right)} \right) + \lambda }}.$$

**SVM:** The SVM algorithm as a classifier and regressor algorithm is applied in ML science. When classifying the data instances, this algorithm uses the hyperplane concept for discriminating the different data on different class labels. This algorithm uses mathematical tricks to classify the other classes by increasing the dataset dimension to higher ones in a pleasant way. Depending on the complexity of the data, the SVM used various Kernel functions, namely, linear, polynomial, radial basis function (RBF), and so on. The RBF (Eq. 8), one of the SVM techniques, is recognized as least square (LS)-SVM having the speed and efficiency in the computation process due to confronting the linear equations [64–66].

8 $$K\left( {x,x^{\prime}} \right) = \exp \left( { - \frac{{\left| {x - x^{\prime}} \right|^{2} }}{{2\sigma^{2} }}} \right).$$

In Eq. 8, |*x* − *x*′| is the square of the Euclidean distance between two input class features, and $\sigma$ is the regularization parameter on training the algorithm.

**MLP:** This feedforward configuration and backpropagation training mode of an Artificial Neural Network (ANN) has many applications in different fields. This ANN type consists of the input, hidden, and output layers. The input layer is responsible for gaining information from the external environment and converting the signals, data, or other input types to the specified calculation formula. The number of nodes in this layer is equal to the study inputs. The second layer is for calculation, so most of the calculation process occurs in this layer. The output layer produces the calculation results and provides us with the ANN’s prediction results. Also, this algorithm uses the activation function for data transformation [67–70].

**J-48:** The J-48 decision tree algorithm, as a newer version of ID3, provided more capability with high flexibility. This decision tree type uses the concept of entropy (Eq. 9) used to split the tree; in other words, the attribute having highly different entropy and the capability of discriminating the various classes from others is considered the node for breaking the tree. By viewing the *x* as an attribute, *p* and *j* are equal to the element and its position; the entropy can be evaluated below:

9 $${\text{Entropy}}\;\left( x \right) = \mathop \sum \limits_{j = 1}^{k} P_{j} \log_{2} \frac{1}{{p_{j} }}.$$

The amount of entropy means the random status of the attribute; in other words, when entropy increases, the random degree is augmented, and decreasing the entropy pertains to the less occasionally, which is suitable for splitting [71–74].

**NB:** The NB (Eq. 10) as a probabilistic algorithm is a commonly used supervised ML algorithm for its high performance. The logic of this algorithm type is that each input variable can independently predict the output class occurrence; in other words, the relationships between the input variables are independently compared to the LR, in which the combinational relationships are considered for forecasting the output class. This algorithm can be considered a simple ML algorithm with high accuracy because of its dependent nature. Some advantages of this algorithm are simplicity in classifying the samples, best classification in the independent mode of variables, and high performance concerning classified inputs [75–78].

10 $$P\left( {C_{{\text{k}}} |x} \right) = \frac{{P\left( {C_{{\text{k}}} } \right)*P(x|C_{{\text{k}}} )}}{P\left( x \right)}.$$

In Formula 10, $P(C_{{\text{k}}} |x)$ is the probability of $C_{{\text{k}}}$ occurrence when having the *x* features with specific values. $PC_{{\text{k}}}$ is the occurrence of the $C_{{\text{k}}}$ class, and $P(x|C_{{\text{k}}} )$ is the probability of the *x* when the class is determined as $C_{{\text{k}}}$.

### *K*-fold cross-validation

In ML, we typically need to split our available data into two sets: a training set and a test set. *K*-fold cross-validation is a technique used to evaluate the performance of an ML model. It involves splitting the available data into *k* equally sized subsets, or “folds.” We then train and evaluate our model *k* times, each time using a different fold as the test set and the remaining folds as the training set. This allows us to get a more reliable estimate of the model’s performance, as we are testing on a different subset of the data each time. To perform *k*-fold cross-validation, we first randomly shuffle the data and then split it into *k* equally sized subsets. We then train our model *k* times, each time using a different fold as the test set and the remaining (*k* − 1) folds as the training set. After each training iteration, we evaluate the model’s performance on the test set and record the performance metric (such as accuracy or mean squared error). Finally, we compute the average performance across all *k* folds to get an estimate of the model’s overall performance. The value of *K* in this research was considered equal to 5.

### Evaluation of the performance of ML algorithms

In this step, we evaluated and compared the selected ML algorithms using the confusion matrix (Table 5) and calculated different performance criteria, including sensitivity, specificity, accuracy, and *F*-score to get the most common algorithm for determining SA. In Table 5, TP and TN are the successful and unsuccessful cases correctly classified by the algorithm, while FN and FP are successful and unsuccessful cases incorrectly classified by the model. Based on the confusion matrix, we calculated the sensitivity (Eq. 11), specificity (Eq. 12), accuracy (Eq. 13), and *F*-Score (Eq. 14) of all ML algorithms. Also, the AUC-ROC diagram of all algorithms was drawn and compared. The k-fold cross-validation (k=10) was considered for measuring errors during the training process. Finally, the most common data mining algorithm for determining the SA was obtained.

11 $${\text{Sesitivity}} = \frac{{{\text{TP}}}}{{{\text{TP}} + {\text{FN}}}},$$

12 $${\text{Specificity}} = \frac{{{\text{TN}}}}{{{\text{FP}} + {\text{TN}}}},$$

13 $${\text{Accuracy}} = \frac{{{\text{TP}} + {\text{TN}}}}{{{\text{TP}} + {\text{FP}} + {\text{FN}} + {\text{TN}}}},$$

14 $$F{\text{-Score}} = \frac{{2{\text{TP}}}}{{2{\text{TP}} + {\text{FP}} + {\text{FN}}}}.$$

**Table 5:** Confusion matrix

Real cases	Predicted cases	
	+	−
+	True positive	False positive
−	False negative	True negative

## Data Availability

The datasets used and/or analyzed during the current study are available from the corresponding author upon reasonable request.
